# Cilia regeneration requires an RNA splicing factor from the ciliary base

**DOI:** 10.1186/s13619-022-00130-x

**Published:** 2022-10-01

**Authors:** Kaiming Xu, Guangshuo Ou

**Affiliations:** Tsinghua-Peking Center for Life Sciences, Beijing Frontier Research Center for Biological Structure, McGovern Institute for Brain Research, School of Life Sciences and MOE Key Laboratory for Protein Science, Tsinghua University, Beijing, China

**Keywords:** PRP-8/PRPF8, RNA splicing factor, Ciliogenesis, Cilium regeneration

## Abstract

Cilia are microtubule-based organelles projected from most eukaryotic cell surfaces performing cell motility and signaling. Several previously recognized non-ciliary proteins play crucial roles in cilium formation and function. Here, we provide additional evidence that the *Caenorhabditis elegans* RNA splicing factor PRP-8/PRPF8 regulates ciliogenesis and regeneration from the ciliary base. Live imaging of GFP knock-in animals reveals that the endogenous PRP-8 localizes in the nuclei and the ciliary base. A weak loss-of-function allele of *prp-8* affects ciliary structure but with little impact on RNA splicing. Conditional degradation of PRP-8 within ciliated sensory neurons showed its direct and specific roles in cilium formation. Notably, the penetrance of ciliary defects correlates with the reduction of PRP-8 at the ciliary base but not nuclei, and sensory neurons regenerated cilia accompanying PRP-8 recovery from the ciliary base rather than the nuclei. We suggest that PRP-8 at the ciliary base contributes to cilium formation and regeneration.

**Supplementary Information:**

The online version contains supplementary material available at 10.1186/s13619-022-00130-x.

## Background

**Fig. 1:**
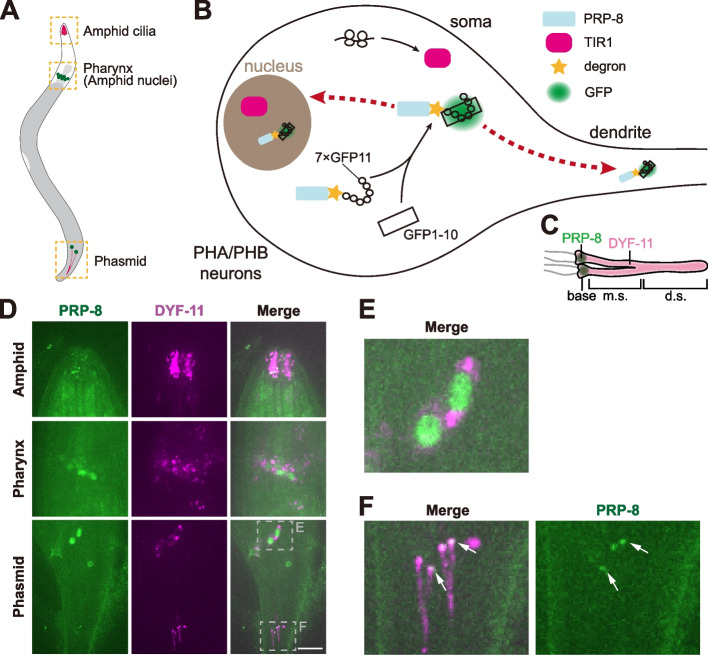
The localization of PRP-8 in the ciliated neurons of *C. elegans.* **A** A cartoon model of the nematode *C. elegans*. Amphid cilia, amphid nuclei, and phasmids are indicated in yellow boxes. **B** Schematic model of the auxin-induced degradation system in the absence of auxin (or IAA, indole acetic acid). Spliceosomal component PRP-8, labeled with degron sequence, can not be targeted to degradation in the phasmids of *C. elegans*. GFP11 can combine with GFP1–10 and therefore mark PRP-8 with green fluorescence, specifically in sensory neurons. No GFP signal can be detected in other tissues, as GFP1–10 is expressed under the control of the ciliated neuron-specific P*che-3* promotor. **C** PRP-8 localizes at the ciliary base. The full-length cilia are visualized by IFT particle marker DYF-11::wrmScarlet. m.s., middle segment; d.s., distal segment. **D** Localization of PRP-8 (labeled with GFP, green) or ciliary marker DYF-11 (labeled with wrmScarlet, magenta) in the amphids (upper and middle) or phasmids (lower). In the absence of auxin, PRP-8 in sensory neurons localizes both in the nuclei (**E**) and at the ciliary bases (**F**). Scale bar, 10 μm. **E** Enlarged phasmid nuclei indicated in (**D**). **F** Enlarged phasmid cilia indicated in (**D**). Ciliary bases are indicated by white arrows

Cilia are microtubule-based structures protruding from the surface of most eukaryotic cells (Anvarian, Mykytyn et al. 2019). The nematode ﻿*Caenorhabditis elegans* has been a valuable system for studying cilium formation and function: 60 of 302 *C. elegans* neurons possess sensory cilia at their dendritic endings (Inglis, Ou et al. 2007, Ward, Thomson et al. 1975, Ware, Clark et al. 1975). In *C. elegans* hermaphrodite, a pair of primary chemosensory organs termed amphids in the head include ciliated neurons whose cell bodies are located ahead of the pharyngeal bulb, and dendritic cilia extend through a channel created by socket cells around the lips (Inglis, Ou et al. 2007). Similar to amphids, the phasmids in the tail contain two ciliated neurons named “PHA” and “PHB,” whose cilia enter the single channel to perceive environmental cues (Fig. 1A) (Inglis, Ou et al. 2007).

Starting from the transition zone, the so-called “middle segment” consists of nine pairs of doublet microtubules generated from the centriole-derived basal body, and the “distal segment” is built of singlet microtubules extended from the A-tubule in the “middle segment” (Blacque and Sanders 2014, Inglis, Ou et al. 2007, Pedersen, Veland et al. 2008). Cilium formation requires multiple biological processes, including intraflagellar transport (IFT), ferrying ciliary cargo molecules from the ciliary base to the ciliary tip, where they incorporate to build cilia, and protein phosphorylation, regulating IFT and ciliary length (Cao, Li et al. 2009, Li, Liu et al. 2021, Wang and Barr 2018, Yuan, Serra et al. 2015). Ciliary dysfunction has been associated with more than 35 types of human disorders, collectively called ciliopathies (Reiter and Leroux 2017).

In addition to the well-characterized ciliary proteins, several family proteins, initially identified as key factors regulating biological processes irrelevant to cilia, have been directly implicated in cilium formation from the ciliary base (Johnson and Malicki 2019). For example, the cytokinetic protein septin SEPT2 localizes at the ciliary base and serves as a diffusion barrier, essential for Sonic hedgehog signal transduction in primary cilia (Hu, Milenkovic et al. 2010). Nuclear import protein importin-β2 regulates the entry of kinesin-2 motor KIF17 into cilia in a small GTPase Ran-dependent manner (Dishinger, Kee et al. 2010), indicating a similar shuttling mechanism of cytoplasmic proteins into the nuclei and the primary cilia. Equally intriguing is the ciliary base localization of pre-mRNA processing factors (PRPFs) PRPF6 / 8 / 31 and splicing factor SNRNP200, implying their potential extranuclear functions (Wheway, Schmidts et al. 2015).

Most newly synthesized pre-mRNAs undergo one or more forms of alternative splicing (AS) by the megadalton spliceosome in the nuclei to form mature mRNAs (Baralle and Giudice 2017, Ule and Blencowe 2019), contributing to cell lineage determination and organ development (Buskin, Zhu et al. 2018). Many RNA splicing factors also localize in the cytoplasm and possess non-splicing functions, such as translation regulation (Haward, Maslon et al. 2021, Sanford, Ellis et al. 2005). PRPF6 / 8 / 31 and SNRNP200 are the critical components of U4 / U6.U5 tri-snRNP in spliceosomes; and their mutations are closely associated with retinitis pigmentosa (RP) (Maxwell, O’Keefe et al. 2021, Yang, Georgiou et al. 2021), a highly variable disorder with the degeneration of retinal cilia and loss of retinal pigment epithelium (Hartong, Berson et al. 2006, Ran and Zhou 2020). Although defective PRPFs may impair pre-mRNA splicing in RP models (Buskin, Zhu et al. 2018, Tanackovic, Ransijn et al. 2011), it remains unresolved whether and how splicing factors play direct roles from the ciliary base to regulating ciliary structure and function.

Here, we show that the endogenous pre-mRNA processing factor PRP-8 / PRPF8 localizes at the ciliary base in *C. elegans* sensory neurons. Using an auxin-induced degradation (AID) system (Zhang, Ward et al. 2015), we find that PRP-8 is crucial for cilia maintenance. By following ciliary regeneration, we observe a successful ciliary reformation when PRP-8 is restored at the ciliary base but not in nuclei, which suggests that PRP-8 at the ciliary base promotes cilia regeneration.

## Results

### *Prp-8 (rr40)* weak loss-of-function allele has a minor effect on RNA splicing

Pre-mRNA processing factor PRP-8 / PRPF8 was identified as the causative gene in RP13 (Greenberg, Goliath et al. 1994). A *C. elegans* genetic suppressor screen identified that the second-last donor splice site of *prp-8* was mutated to AT in *prp-8 (rr40)* allele (Hebeisen, Drysdale et al. 2008), resulting in failed excision of the second-last intron in *prp-8* pre-mRNA (Fig. S1A). Using the CRISPR-Cas9-based genome editing method (Cong, Ran et al. 2013, Nance and Frokjaer-Jensen 2019), we introduced the same mutation into the wild-type (WT) N2 strain background. We also constructed RP11^A225P^ -associated mutation in *C. elegans*, corresponding to ﻿PRPF31^A216P^ variant detected in retinitis pigmentosa patients (Fig. S1B) (Maxwell, O’Keefe et al. 2021).

Demonstrated as a weak loss-of-function allele (Hebeisen, Drysdale et al. 2008), *prp-8 (rr40)* is viable and plays a limiting role in regulating splicing efficiency in a germ-line-specific manner. It was controversial whether PRPF31^A216P^ affects splicing or not (Buskin, Zhu et al. 2018, Deery, Vithana et al. 2002). Thus, we applied the established iREAD pipeline to evaluate the overall splicing efficiency in *prp-8 (rr40)* and PRPF31^A216P^ (Fig. S1C) (Li, Funk et al. 2020). *plk-1 (syb4603)* allele, containing a T194D hyperactive mutation of POLO kinase but unrelated to splicing, serves as a negative control (Lee and Erikson 1997). RNA interference (RNAi) of Sm core protein SNR-6 (Sm E in yeast) is a positive control for evaluating impaired RNA splicing. We only detected 5, 8, and 5 unspliced independent introns in *prp-8 (rr40)*, *prp-31*^A225P^, and *plk-1*^T194D^ mutant animals, respectively. By contrast, *snr-6* RNAi animals had 27 unspliced independent introns (Fig. S1C). We detected the failed excision of the second-last intron of *prp-8* in *rr40* mutant, and RNA-seq reads covering the first intron of *snr-6* were seen as a “fake” unspliced event in *snr-6* RNAi animals (Fig. S1D), validating our bioinformatic analyses. Although partial ciliogenesis defect has been reported in *rr40* mutants (Wheway, Schmidts et al. 2015), the unspliced introns in this allele were not known to be associated with cilia-related genes (Fig. S1D). These results indicate that *prp-8 (rr40)* allele does not generate a major impact on RNA splicing efficiency and suggest that PRP-8 may, at least, have partial splicing-independent roles in ciliogenesis.

### PRP-8 conditional degradation causes ciliary defects

Because severe loss of function of PRP-8 led to embryonic lethality (Sonnichsen, Koski et al. 2005), we attempted to use the auxin-induced degradation (AID) system to achieve PRP-8 conditional degradation (Zhang, Ward et al. 2015). The method includes the Skp1-Cullin-F-box (SCF) E3 ubiquitin ligase subunit TIR1 responsible for recognizing substrates, the TIR1 recognizing sequence degron, and the auxin (indole-3-acetic acid). We first constructed four knock-in or transgenic strains (Fig. S2A). We knocked the degron sequence along with seven tandem GFP11 into the C-terminal of endogenous PRP-8 in WT animals, and we then knocked the *T2A-GFP1–10* sequence into the 3′-terminal of the ciliated neuron-specific dynein-2 heavy chain *che-3* sequence (Cabantous, Terwilliger et al. 2005, Goudeau, Sharp et al. 2021). The T2A self-split sequence ensures the separation of CHE-3 and GFP1–10, which enables a specific GFP labeling of PRP-8 in ciliated neurons. We knocked a red fluorescence protein wrmScarlet into the C-terminal of endogenous IFT complex B subunit DYF-11 to label the sensory cilia (Goudeau, Sharp et al. 2021). In addition, a single copy of *tir1*, is expressed under the ciliated neuron-specific promotor P*dyf-1* (Obinata, Sugimoto et al. 2018), allowing a specific degradation of PRP-8 in these neurons without affecting other tissues. We combined these elements by the genetic cross (Fig. S2 A and B), and we also generated a negative control strain, GOU4755, that does not express TIR1.

**Fig. 2:**
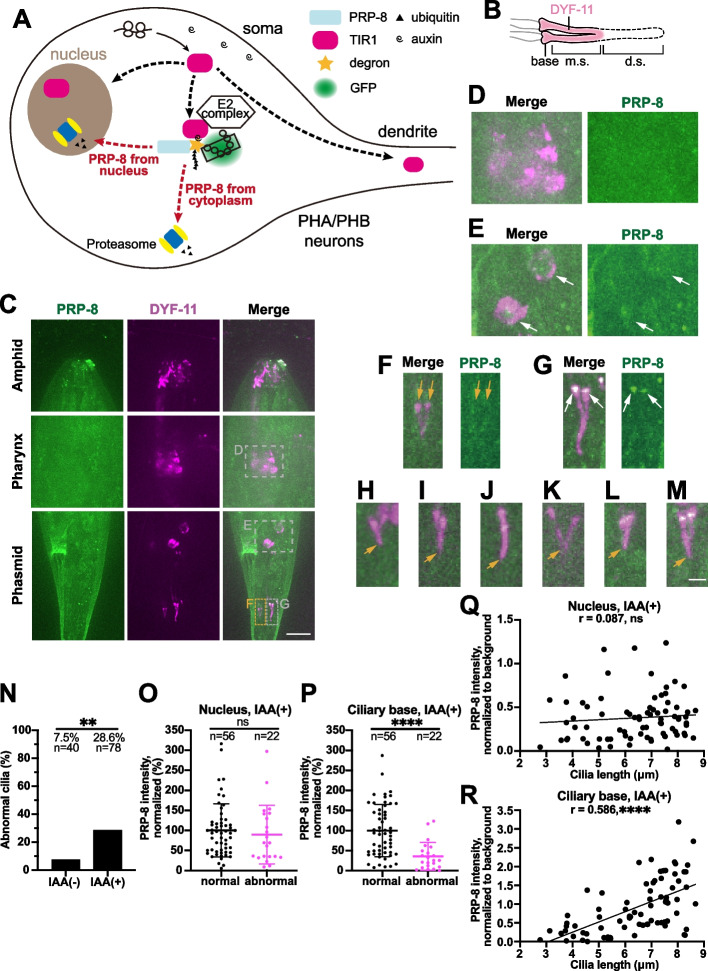
Auxin-induced degradation of PRP-8. **A** Schematic model of the auxin-induced degradation system in the presence of 4 mM auxin. Transgenic E3 ubiquitin ligase TIR1 can bind degron-tagged PRP-8 and label this protein with ubiquitin (Ub) chains. Polyubiquitinated PRP-8 is degraded by the proteasome. Degron-tagged PRP-8 in other tissues remains stable because transgenic TIR1 (single copy) is expressed under the control of the ciliated neuron-specific P*dyf-1* promotor. **B** PRP-8 at the ciliary base is degraded in the presence of 4 mM auxin. Defective cilia (e.g. without distal segments) can be seen when visualized with a DYF-11::wrmScarlet marker. m.s., middle segment; d.s., distal segment. **C** Localization of PRP-8 in the presence of 4 mM auxin. The positions of amphid (**D**) or phasmid (**E**) nuclei are indicated in gray boxes. Phasmid cilia are indicated in yellow (**F**) or gray (**G**) box. Scale bar, 10 μm. **D** Enlarged amphid nuclei indicated in (**C**). **E** Enlarged phasmid nuclei indicated in (**C**). Nuclei are indicated by white arrows. **F** Enlarged phasmid cilia indicated in yellow box in (**C**). Ciliary bases are indicated by yellow arrows. **G** Enlarged phasmid cilia indicated in gray box in (**C**). Ciliary bases are indicated by white arrows. **H**-**M** Representative images of defective phasmid cilia in the presence of 4 mM auxin. Ciliary tips are indicated by yellow arrows. Scale bar, 2 μm. **N** Percentage of abnormal cilia in the two groups. IAA (+) group was treated with 4 mM auxin on NGM plates for 24 hours. IAA (−) group was not treated with auxin on NGM plates for 24 hours. For each worm, two cilia from different phasmids are counted. *n* indicates the number of cilia. **P < 0.01 via Fisher’s exact test. **O** Quantification of nuclear PRP-8 intensity in IAA (+) group. Normal or abnormal cilia correspond to those in (**N**). The mean intensity of normal group is normalized to 100%. *n* indicates the number of nuclei. **P** Quantification of ciliary basal PRP-8 intensity in IAA (+) group. Normal or abnormal cilia correspond to those in (**N**). The mean intensity of normal group is normalized to 100%. *n* indicates the number of cilia. ﻿**P < 0.01 and ****P < 0.0001 and ns denotes *P* > 0.05 via unpaired *t-*test. ﻿Error bars, SD. **Q**-**R** Scatterplot of each cilium in IAA (+) group. The length of cilia with their nuclear PRP-8 intensities are plotted in (**Q**). The length of cilia with their ciliary basal PRP-8 intensities are plotted in (R). *r* indicates the Pearson correlation coefficient. ****P < 0.0001 and ns denotes *P* > 0.05 via paired *t-*test

In agreement with the previous immunofluorescence results in mammalian cells (Wheway, Schmidts et al. 2015), the PRP-8-degron-7XGFP11 fusion protein directly binds soluble GFP1–10 localizes in nuclei and at the ciliary base (Fig. 1A-F). We evaluated the efficiency of PRP-8 degradation in phasmid neurons: PRP-8 loss was observed as early as 2 h since auxin treatment. In the presence of auxin (Fig. 2A-B), we found a significant loss of PRP-8 from the nuclei. By normalizing the PRP-8 signal with background (Fig. S3A), we showed the degradation follows a reverse logistic curve (Fig. S3B-C). After 6-hour auxin treatment, the PRP-8 fluorescence was only 0.2-fold higher than the background, markedly lower than 1-fold higher in the mean without auxin treatment, which indicates that the AID system removed 80% of nuclear PRP-8. However, we did not observe a significant loss of PRP-8 at the ciliary base and ciliary defect after auxin treatment for 8 hours (Fig. S3D). For the negative control strain, both nuclear and ciliary basal PRP-8 in the amphid and phasmid neurons remained constant before or after 4 mM auxin treatment for 24 hours (Fig. S4A-E). We did not detect apparent ciliary defect after auxin treatment (Fig. S4F), which indicates that 4 mM auxin treatment is not deleterious to PRP-8 and cilia in *C. elegans*.

After AID-induced PRP-8 degradation for 24 hours, we detected ciliary defects with an occurrence rate of 28.6% (22 out of 78 examined phasmid cilia pairs) (Fig. 2C-N). Seven of these abnormal cilia lost both middle and distal ciliary segments (Fig. S3E). In the rest 15, the length of their distal ciliary segments was shorter than 1.5 μm, indicating the reduced distal segments (Fig. S3F). In these defective cilia, we found a significant loss of PRP-8 at the ciliary base. We noticed that 71.4% of cilia appeared normal, with a higher PRP-8 level at the ciliary base (Fig. 2G). We calculated nuclear PRP-8 intensities, basal PRP-8 intensities, and cilia length (Fig. S5A) in phasmid neurons having intact or short cilia, respectively. While nuclear PRP-8 intensities are indistinguishable between the two groups (Fig. 2O; Fig. S5B), ciliary PRP-8 intensities in the short cilia group are much lower than those in the normal group (Fig. 2P; Fig. S5C), implying a correlation between ciliary defects and the ciliary but not nuclear PRP-8. To further demonstrate this correlation, we plotted the length of all the individual cilia with their corresponding nuclear PRP-8 (Fig. 2Q) or ciliary PRP-8 (Fig. 2R) intensities. The nuclear PRP-8 does not correlate with the ciliary length (Correlation efficiency = 0.087); however, the ciliary basal PRP-8 exhibits a significantly positive correlation with the ciliary length (Correlation efficiency = 0.586), suggesting a role of PRP-8 at the ciliary base in ciliogenesis.

We further dissected the role of the nuclear and ciliary basal PRP-8 in cilium formation and maintenance. By normalizing PRP-8 intensities with background (Fig. S3A), we divided cilia into two groups: PRP-8 (+) group with PRP-8 intensity > 0.5 and PRP-8 (−) group with PRP-8 intensity < 0.3. We observed 36.8% and 34.3% ciliary defects in the nuclear PRP-8 (+) group and the nuclear PRP-8 (−) group, respectively (Fig. S5D), and ciliary length was not different between these two groups (Fig. S5E). However, for the PRP-8 at the ciliary bases, 66.7% of ciliary defects were observed in the ciliary basal PRP-8 (−) group, which is significantly higher than those from the ciliary basal PRP-8 (+) group (Fig. S5F). Consistently, the ciliary PRP-8 (−) group formed much shorter cilia than those in the ciliary PRP-8 (+) group (Fig. S5G). These data provide evidence for a robust correlation between the normal ciliary structure and the presence of PRP-8 at the ciliary base.

### Restoration of PRP-8 at the ciliary base but not nuclei promotes cilia regeneration

**Fig. 3:**
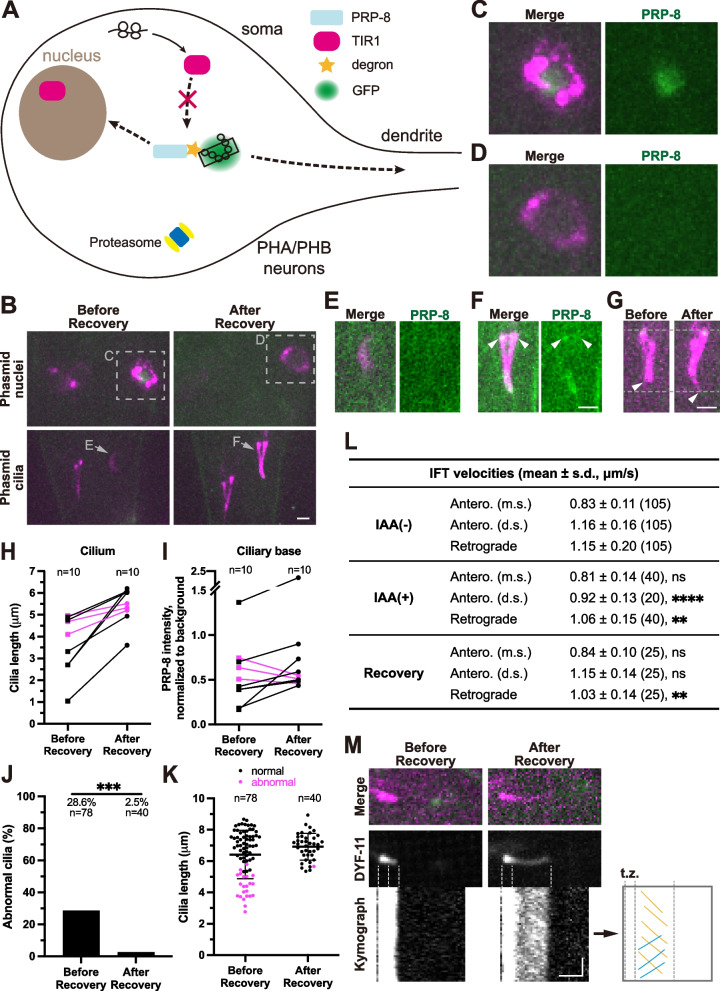
Restoration of PRP-8 and the regeneration of cilia. **A** Schematic model of the restoration of PRP-8. TIR1 no longer binds degron-tagged PRP-8 when auxin is removed. Thus newly synthesized PRP-8 is not degraded by the proteasome. **B** Localization of PRP-8 in the phasmids before or after removal of auxin for 24 hours. The worm was treated with 4 mM auxin on NGM plates for 24 hours before removal of auxin. Phasmid nuclei are indicated in gray boxes (**C**-**D**). Phasmid cilia are indicated in gray arrows (**E**-**F**). Scale bar, 2 μm. **C** Enlarged phasmid nuclei before recovery indicated in (**B**). **D** Enlarged phasmid nuclei after recovery indicated in (B). **E** Enlarged phasmid cilia before recovery indicated in (**B**). **F** Enlarged phasmid cilia after recovery indicated in (**B**). Ciliary bases are indicated by white arrowheads. Scale bar, 2 μm. **G** Representative images of regenerated cilia after recovery for 24 hours. The same cilia from the same worm are compared. Ciliary tips are indicated by white arrowheads. Scale bar, 2 μm. **H** Cilia length before or after removal of auxin for 24 hours. 10 regenerated cilia are counted. **I** Ciliary basal PRP-8 intensities before or after removal of auxin for 24 hours. 10 regenerated cilia are counted. The three cilia with decreased ciliary PRP-8 intensities after recovery are indicated in magenta in (**H**) and (**I**). **J** Percentage of abnormal cilia before or after removal of auxin for 48 hours. For each worm, two cilia from different phasmids are counted. *n* indicates the number of cilia. ***P < 0.001 via Fisher’s exact test. **K** Quantification of cilia length before or after removal of auxin for 48 hours. Normal or abnormal cilia correspond to those in (**J**). *n* indicates the number of cilia. **L** Summary of IFT velocities. Numbers of IFT particles are shown in the brackets. IAA (−) group, before auxin treatment; IAA (+) group, after auxin treatment for 24 hours; Recovery group, after recovery for 24 hours; Antero., anterograde; m.s., middle segment; d.s., distal segment. **P < 0.01 and ****P < 0.0001 and ns denotes *P* > 0.05 via unpaired *t-*test. **M** Kymographs of cilia before or after removal of auxin for 24 hours. Anterograde IFT particles are marked with yellow lines. Retrograde IFT particles are marked with blue lines. t.z., transition zone. Scale bar (horizontal), 2 μm; Scale bar (vertical), 2 s

Considering that auxin wash disassembles the AID system and recovers the target protein (Zhang, Ward et al. 2015), we next examined whether restoration of PRP-8 promotes cilia regeneration. By transferring auxin-treated animals with ciliary defects to NGM plates without auxin (Fig. 3A; Fig. S2C), we found that 45.5% of animals (10 of 22) regenerated their cilia after 24 hours, among which seven restored apparent PRP-8 fluorescence at the ciliary bases (Fig. 3B-I; Fig. S5H-I). The rest of the three regenerated cilia slightly increased their ciliary length with lower PRP-8 intensities than those regenerated longer cilia (Fig. 3H-I). Next, we evaluated the overall regeneration of cilia after recovery for 48 hours (Fig. 3J-K). The only defective cilia after 48-hour recovery were devoid of ciliary PRP-8 intensities (Fig. 3K; Fig. S5J-L), further supporting that PRP-8 at the ciliary base contributes to the regeneration of cilia.

**Fig. 4:**
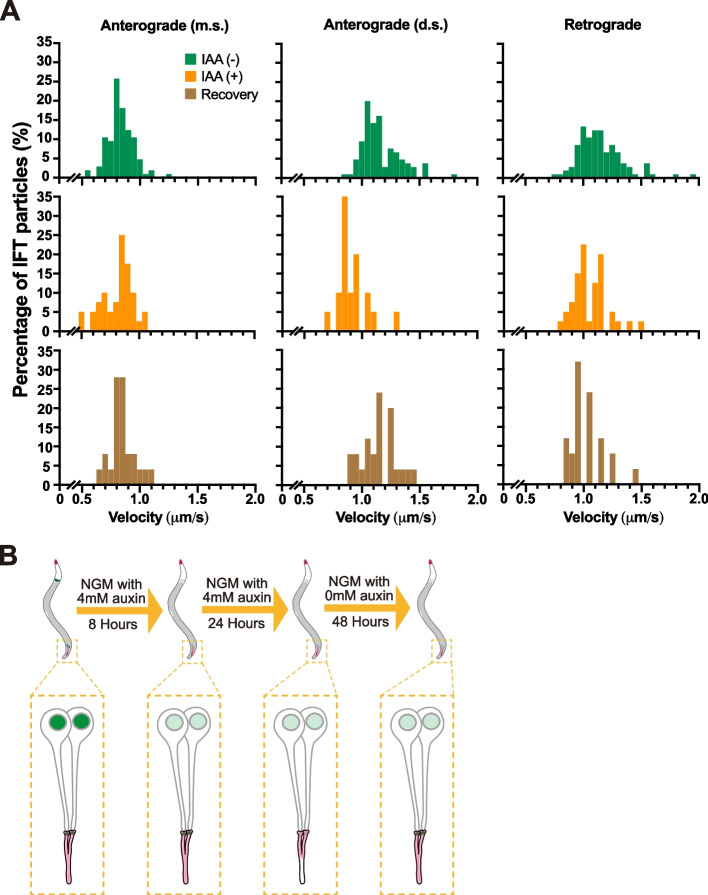
Cilia regeneration and working model. **A** Histograms of IFT velocity. IAA (−), IFT particles in cilia before auxin treatment; IAA (+), IFT particles in defective cilia after 4 mM auxin treatment for 24 hours; Recovery, IFT particles in regenerated cilia which are defective after auxin treatment but regenerated when recovery. m.s., middle segment; d.s., distal segment. **B** A working model illustrating the dependency of ciliogenesis and cilia regeneration on ciliary basal PRP-8 rather than nuclear PRP-8. After 8-hour auxin treatment, PRP-8 in the phasmid nuclei instead of ciliary bases was degraded, and cilia remained intact; After 24-hour auxin treatment, both PRP-8 in the nuclei and at the ciliary bases were degraded, thus ciliary defects could be seen; Further, after 48-hour auxin-free recovery, cilia regenerated along with restoration of ciliary PRP-8 rather than nuclear PRP-8

Both ciliogenesis and cilia regeneration depend heavily on intraflagellar transport (IFT) (Nakayama and Katoh 2020, Wang, Jack et al. 2021). We finally investigated the dynamics of cilia regeneration at IFT level. By measuring the speeds of IFT particles in these regenerated cilia (Fig. 3L-M), we found that IFT, albeit slower in the distal segment in the anterograde direction (Ou, Blacque et al. 2005), recovered (Antero. m.s.: 0.84 μm/s; Antero. d.s.: 1.15 μm/s; Retrograde: 1.03 μm/s in mean), consistent with the notion that bidirectional IFT drives cilia formation and regeneration (Fig. 3L; Fig. 4A). Strikingly, in the animals which regenerated their cilia and restored the PRP-8 signal at the ciliary base, we failed to detect any restoration of PRP-8 fluorescence in the nuclei after 24-hour or even 48-hour recovery (*N* > 30 animals, Fig. 3B-D; Fig. S5H-K).

## Discussion

This study provides evidence that the RNA splicing factor PRP-8 regulates cilium formation and regeneration at the ciliary base. Live imaging of GFP knock-in animals illustrates the endogenous localization pattern of PRP-8 in nuclei and at the ciliary base, which is consistent with the immunofluorescence results from fixed mammalian cells (Wheway, Schmidts et al. 2015). Conditional degradation of PRP-8 revealed its roles in cilium formation within ciliated sensory neurons, excluding the possibility that RNA splicing defects in other tissues caused side effects on cilia. Notably, the penetrance of ciliary defects correlates with the reduction of PRP-8 at the ciliary bases but not nuclei; and the sensory neurons regenerated their sensory cilia accompanying a specific recovery of PRP-8 from the ciliary base rather than nuclei, further supporting the hypothesis that PRP-8 at the ciliary base plays crucial roles on cilia formation (Fig. 4 B). We provided evidence for the positive correlation between ciliary basal PRP-8 and ciliary morphology (Fig. 2R), and we did not detect any apparent correlation between nuclear PRP-8 and ciliogenesis (Fig. 2Q), which suggests that degradation of nuclear PRP-8 may have a minor impact on cilia. However, such correlations cannot exclude the possibility that RNA splicing is involved in cilia formation and maintenance. Considering that almost all the ciliary genes consist of multiple exons, we argue that the RNA splicing function of PRP-8 is essential for ciliogenesis. Although we did not detect a noticeable recovery of PRP-8 fluorescence in the nuclei from animals regenerating cilia after auxin removal, which may be due to the nuclear enrichment of TIR1 (Watanabe, Mano et al. 2016), we speculate that the existing spliced RNA in the cytosol may be sufficient to express ciliary proteins to reform cilia. Alternatively, a small amount of nascent PRP-8, beyond the detection of our microscopic limit, may support RNA splicing in the nuclei. On the other hand, we only detected 27 unspliced introns in *snr-6* RNAi animals using the established pipeline “iREAD”. Because *snr-6* or *prp-8* RNAi induces sterility and lethality in worms, and their null alleles are not viable, we speculate that a complete loss of *snr-6* or *prp-8* must cause more severe defects in RNA splicing.

With auxin treatment, we noticed that some worms had strong ciliary PRP-8, which may be resulted from the less efficiency of auxin treatment in these animals. Considering that PRP-8 was gradually degraded, some worms may have more spliced RNAs than others, some of which may encode ciliary genes. These worms can have more PRP-8 and ciliary proteins, generating longer cilia or regenerating faster. Thus, the future study needs to develop a methodology to block the PRP-8 function at the ciliary base, determining the specific role of the ciliary PRP-8 in this process. Neither can we rule out the possibility that the amount of PRP-8 at the ciliary base can be a concomitant event with abnormal ciliogenesis, despite those control experiments (Fig. 1D-F; Fig. S4A-F).

How does PRP-8 act from the ciliary base to promote cilium formation and regeneration? Recent studies reported that PRPF8 functions as the receptor of linear ubiquitin chains at the ciliary base to facilitate CP110-CEP97 complex removal at the initial stage of ciliogenesis (Liu, Zhang et al. 2021, Shen, Yuan et al. 2022). Such a role of PRPF8 may apply to cilium regeneration from the existing middle ciliary segment, which suggests that PRPF8 must play additional roles from the ciliary base. In line with this idea, the CP110-CEP97 complex has not been described in *C. elegans*. Proteins at the ciliary base generally promote the entry of ciliary precursors or help assemble or activate IFT machinery. Thus, it is tempting to speculate that PRP-8 might be involved in these events to promote cilium formation and regeneration.

## Conclusions

In summary, our study provided evidence that splicing factor PRP-8/PRPF8 at the ciliary base regulates ciliogenesis and cilia regeneration in the nematode *C. elegans*. *Prp-8 (rr40)* weak loss-of-function allele caused ciliary defects but had minor effect on splicing efficiency. This study used the ciliated neuron-specific AID method to degrade PRP-8 in the amphid and phasmid neurons. Notably, the penetrance of ciliary defects correlates with the degradation of PRP-8 at the ciliary base but not the nuclei. Consistently, the sensory neurons regenerated cilia accompanying PRP-8 recovery from the ciliary base rather than the nuclei, implying direct and specific roles of ciliary PRP-8 in cilium formation.

## Methods

### Genome editing

We used CRISPR-Cas9-mediated genome editing to create multiple knock-in strains (Cong, Ran et al. 2013). ﻿The CRISPR design tool (https://zlab.bio/guide-design-resources. Accessed 10 Jun 2022.) selected the target sequence. The sgRNA sequences were inserted into the pDD162 vector (Addgene #47549) by linearizing this vector with 15 bp-overlapped primers. The resulting PCR products were treated with DpnI digestion overnight and transformed into *E. coli* Trans5α. The linearized PCR products were cyclized by spontaneous recombination in bacteria. For degron sequence or fluorescence tag knock-in, homology recombination (HR) templates were constructed by cloning the homology arms into pPD95.77 plasmids using the In-Fusion Advantage PCR cloning kit (Clontech, #639621). Subsequently, target sequences were inserted into the constructs with a flexible linker before the stop codons. Target sites in the templates were modified with synonymous mutations. CRISPR-Cas9 constructs and HR templates were purified with AxyPrep Plasmid Purification Miniprep Kit (Axygen, #AP-MN-P-250) and PureLink Quick PCR purification Kit (Invitrogen, #K310001) and co-injected into the gonads of young adult worms at 50 ng/μl with the *rol-6 (su1006)* and P*odr-1::dsRed* selection markers. P*dyf-1::tir1* was inserted into pOB4 HR templates targeting + 0.77 cM on chromosome II (Obinata, Sugimoto et al. 2018). F1 transgenic progenies were singled and screened by PCR. SunyBiotech Ltd. generated some strains and alleles, the name of these strains started with ‘PHX’, and the names of these alleles started with ‘*syb*’ (Fig. S2B; Table S1). Other strains were created by ourselves; their names began with ‘*cas*’ (Fig. S2B; Table S1).

### Strains, culture, and genetic cross

*C. elegans* were cultured at 20 °C on standard nematode growth medium (NGM) plates seeded with the ﻿*Escherichia coli* OP50 strain unless described otherwise. The Wild-Type strain was Bristol N2, and some strains were provided by the *Caenorhabditis* Genetics Center (CGC). Other strains were created using the CRISPR-Cas9-mediated genome editing method. 20 μl suspended OP50 was dropped at the center of NGM plates to make cross plates. Ten males carrying *him-5 (e1490)* allele and five hermaphrodites were transferred to this plate for 24 hours. F1 and F2 hybrid progenies were singled and screened by PCR.

### Auxin treatment

Auxin treatment was performed by transferring Day-1 adult hermaphrodites to bacteria-seeded plates containing auxin. The natural auxin indole-3-acetic acid (IAA) was purchased from Alfa Aesar (#A10556). A 400 mM stock solution in ethanol was prepared and was stored at 4 °C for up to 1 week. Auxin was diluted into the NGM agar at 4 mM before pouring the plates. Because 4 mM auxin inhibited bacterial growth (Zhang, Ward et al. 2015), a fresh OP50 culture with 4 mM auxin was concentrated for 4 folds before spreading plates. Plates could be saved at 4 °C for up to 2 days. For the recovery assay, worms on auxin-enriched plates were transferred to NGM plates without any auxin to permit spontaneous restoration. If necessary, anesthetized worms mounted on agarose pads were picked carefully to NGM plates and washed 3 times with M9 buffer, and only viable worms were used for further imaging. The same cilia before or after recovery were determined according to their relative positions in the tails of worms. The worms were grown in the dark during auxin treatment or recovery at 20 °C.

### Live cell imaging

Auxin-treated or untreated *C. elegans* hermaphrodites were anesthetized with 0.1 mmol/L levamisole in M9 buffer, mounted on 3% agarose pads, and maintained at 20 °C. Imaging was performed using an Axio Observer Z1 microscope (Carl Zeiss) equipped with 488 and 561 laser lines, a Yokogawa spinning disk head, an Andor iXon+ EM-CCD camera, and a Zeiss 100× / 1.46 objective. ﻿Images were acquired at 0.7-μm z-spacing by μManager (https://www.micro-manager.org. Accessed 10 Jun 2022.) except for measuring IFT velocities. Images were not deconvolved. All the images were taken using identical settings (EM-gain: 250, exposure time: 200 ms, 50% of max laser) except for measuring speeds of intraflagellar transport (30% of max laser). Image analysis and measurement were performed with ImageJ software (http://rsbweb.nih.gov/ij/. Accessed 10 Jun 2022.). Image stacks were z-projected using maximum projection except for Fig. 3M, which used sum projection for better visualization. The visualized color of the L-561 channel was changed from red to colorblind-safe magenta in ImageJ. All images shown were adjusted linearly. Fluorescence quantification was performed on a single, unprocessed (or before z-projection) optical section from the middle of each data stack. To quantify the PRP-8 fluorescence intensity in the nuclei or at the ciliary bases, we measured GFP particle intensity using a plugin (‘Analyze-Measure’ for L-488 channel) in ImageJ subtracted and then divided by background fluorescence (Fig. S3A). To compare GFP intensities between the two groups, we normalized ﻿the fluorescence intensity by dividing each value by the mean intensity of the first group. We used a ciliary marker DYF-11::wrmScarlet to measure the length of cilia and IFT velocities. Stacked images covering the whole cilia structures were used to measure the cilia length. Movies only in stable focal planes that covered the entire cilia structures were used to generate kymographs. A detailed analysis of IFT velocities could be referred to as established protocols (Ou, Blacque et al. 2005).

### Sequence alignment

﻿ Sequence alignment was performed using Clustal X2.1 (http://www.clustal.org/. Accessed 10 Jun 2022.) and Jalview 2.11.2.1. Protein sequences were obtained from Wormbase (http://www.wormbase.org/) or National Center for Biotechnology Information (www.ncbi.nlm.nih.gov/. Accessed 10 Jun 2022.). Sequence IDs used include NP_491527.1 (*Ce*PRP-31), NP_648756.1 (*Dm*Prp31), NP_056444.3 (*Hs*PRPF31), NP_081604.3 (*Mm*Prpf31), NP_011605.1 (*Sc*PRP31). ﻿The full-length sequences were used to perform alignment.

### RNA sequencing

﻿ Synchronized worms were cultured on NGM plates without OP50 bacteria. Worms were harvested at the L1 larval stage and then lysed with TRIzol reagent (Invitrogen). Total RNA was extracted according to the standard manufacturer’s protocol. SUPERase•In™ RNase Inhibitor (Invitrogen) was used in each step to prevent RNA from degradation. RNA concentration was quantified using the Qubit RNA High Sensitivity Assay Kit (Invitrogen). 100 ng of total RNA was used for library preparation using the KAPA RNA HyperPrep Kit (KAPA Biosystems). Library samples were analyzed by Agilent 2100 bioanalyzer system and Qubit for quality control and quantification. The samples were sequenced on an Illumina HiSeq-PE150 platform. A total of 150-bp paired-end reads were generated.

### Splicing efficiency analysis

The raw sequencing reads were trimmed using Trim_galore (version 0.6.4) to remove the low-quality bases and adaptor sequences. After trimming, paired-end reads with at least 20 nucleotides in length were aligned to *C. elegans* reference genome (WBcel235) using STAR (2.7.3a) with the parameter ‘—sjdbOverhang 139’ to acquire BAM-formatted files. Independent intron (intron that does not overlap with any exon) coordinates were extracted using GTFtools (0.8.0) to acquire BED-formatted files. Events of intron retention (IR) were defined using iREAD (https://www.genemine.org/iread.php. Accessed 10 Jun 2022.) (parameter ‘-j 2 -b 50’) with the following criteria (Li, Funk et al. 2020): introns with at least 20 intronic reads and more than 0.9 entropy score (this score measures to which extent reads are uniformly distributed across the whole region for a given intron). Unspliced introns were identified when an IR event only existed in the experimental group rather than the control group (Bristol N2 or worms without RNA interference treatment). We used IGV software (2.11.3) to visualize the distribution of raw reads along the *C. elegans* reference genome.

### RNAi feeding

RNAi bacteria for *snr-6* were grown in LB media containing carbenicillin (100 μg/ml) at 37 °C overnight. The next day, RNAi bacteria were induced with 1 mM isopropyl-β-D-thiogalactopyranoside (IPTG) for another 1 hour at 37 °C and then seeded on NGM plates supplemented with carbenicillin (100 μg/ml) and 1 mM IPTG. Synchronized worms (strain: GOU4596) were transferred to RNAi plates and seeded by *E. coli* HT115 strains expressing an empty vector (EV) as control or double-stranded RNA. RNAi strain was from the Source BioScience Ltd.

### Statistical analysis

﻿ All experiments were repeated at least two times with identical or similar results. ﻿We used GraphPad Prism 9 (GraphPad Software, Inc.) for statistical analyses. Appropriate statistical tests were used for every figure. Unpaired or paired Student’s *t-*tests were performed to compare the mean values between the two groups, and Fisher’s exact tests were performed to judge the correlation between the two variables. Data met the assumptions of the statistical tests described for each figure. Statistical parameters, including the exact value of *n* and descriptive statistics (mean ± SD) and statistical significance, were reported in the figures and the figure legends. Data were judged to be statistically significant when *P* < 0.05 by the two-tailed Student’s *t*-test or Fisher’s exact test. Statistical significances were designated as ns *P* > 0.05, * *P* < 0.05, ** *P* < 0.01, *** *P* < 0.001 and **** *P* < 0.0001, as compared to appropriate controls. Information about statistical tests, *P* values, and *n* numbers were provided in the respective figures and figure legends.

## Supplementary Information

**Additional file 1: Fig. S1.** prp-8 (rr40) weak allele has a minor effect on splicing. **Fig. S2.** Genetic construction of PRP-8 conditional degradation strain. **Fig. S3.** Gradual degradation of PRP-8 in the presence of auxin. **Fig. S4.** PRP-8 remains stable in the absence of TIR1. **Fig. S5.** Statistics of PRP-8 intensities.

**Additional file 2: Table S1. ***C. elegans* strains in this study. **Table S2.** Nucleotide sequences and PCR primers.

## Data Availability

All data is available in the main text or the supplementary materials.

## Abbreviations

*GFP*: Green fluorescence protein
*IFT*: Intraflagellar transport
*PRPF*: Pre-mRNA processing factor
*AS*: Alternative splicing
*RP*: Retinitis Pigmentosa
*AID*: Auxin-induced degradation
*RNAi*: RNA interference
*SCF*: Skp1-Cullin-F-box
*m.s.*: Middle segment
*d.s.*: Distal segment
