# Correction: Cilia regeneration requires an RNA splicing factor from the ciliary base

**DOI:** 10.1186/s13619-022-00147-2

**Published:** 2022-11-09

**Authors:** Kaiming Xu, Guangshuo Ou

**Affiliations:** Tsinghua-Peking Center for Life Sciences, Beijing Frontier Research Center for Biological Structure, McGovern Institute for Brain Research, School of Life Sciences and MOE Key Laboratory for Protein Science, Tsinghua University, Beijing, China

**Correction: Cell Regen 11, 29 (2022)**

**https://doi.org/10.1186/s13619-022-00130-x**

Following publication of the original article (Xu and Ou 2022), it is reported that the supplement figure S1-S5 were missing from the article. Additional file 1: Fig. S1 contains only figure legends but no figures.

The original article (Xu and Ou 2022) has been corrected.
