# Supplementary material for: Cilia regeneration requires an RNA splicing factor from the ciliary base
Source: Cell Regen. 2022 Oct 1;11:29. doi: 10.1186/s13619-022-00130-x (PMC9525525; doi:10.1186/s13619-022-00130-x)
Supplement: Supplementary file 1 — Additional file 1: Fig. S1. prp-8 (rr40) weak allele has a minor effect on splicing. Fig. S2. Genetic construction of PRP-8 conditional degradation strain. Fig. S3. Gradual degradation of PRP-8 in the presence of auxin. Fig. S4. PRP-8 remains stable in the absence of TIR1. Fig. S5. Statistics of PRP-8 intensities. [file 13619_2022_130_MOESM1_ESM.zip › Xu_Figure legend S1-S5.docx]

**Figure S1. *prp-8 (rr40)* weak allele has a minor effect on splicing**

(A) RNA expression profiles of Wild Type animals and *prp-8 (rr40)* allele at *prp-8* locus. g7337a donor splice site mutation is indicated in red. The RNA-seq signals are shown in the range (0-2500).

(B) Multi-sequence alignment of *C. elegans* (Ce) PRP-31 with its homologs in *Drosophila* (Dm), human (Hs), mouse (Mm), and yeast (Sc). The conservatism of each amino acid site is shown below. Red aster shows the RP11 disease-related mutation site.

(C) Unspliced intron numbers in different strains with mutations or RNAi treatment. *prp-8*, *prp-31,* and *snr-6* encode core spliceosomal components. *plk-1* encodes POLO kinase unrelated to splicing and serves as a control group.

(D) List of genes carrying unspliced introns in *prp-8 (rr40)* and *snr-6* RNAi group. *prp-8* and *snr-6* are underscored in blue. One gene may carry more than one unspliced intron.

**Figure S2. Genetic construction of PRP-8 conditional degradation strain**

(A) Transgenic structures of core elements. For *prp-8*, a *degron* short sequence and seven tandem *gfp11* sequences are inserted behind *prp-8* locus; For *che-3*, a *T2A* split-sequence and a *gfp1-10* sequence are inserted behind *che-3* locus; For *dyf-11*, a *wrmScarlet* sequence is inserted behind *dyf-11* locus; *tir-1* expressed under the ciliated neuron-specific promotor P*dyf-1* is inserted at +0.77 cM on chromosome II. pF, forward primer; pR, reverse primer. More details can refer to Table S2.

(B) Image of agarose gel electrophoresis shows that all transgenes are properly inserted into the worm genome.

(C) Flowchart of the auxin-induced degradation of PRP-8. Day-1 adult hermaphrodites were transferred to bacteria-seeded NGM plates containing 4 mM auxin for 24 hours. For the restoration of PRP-8, the treated worms were transferred back to auxin-free NGM plates to permit spontaneous recovery for 24 or 48 hours.

**Figure S3. Gradual degradation of PRP-8 in the presence of auxin**

(A) Quantificational method of PRP-8 intensities normalized to the background.

(B) Gradual degradation of PRP-8 in the nuclei in the presence of 4 mM auxin. Images were taken every 2 hours. Scale bar, 2 μm.

(C) Quantification of nuclear PRP-8 intensity in (B). ***P* < 0.01 via unpaired *t-*test. ﻿Error bars, SD.

(D) Representative image of phasmids after auxin treatment for 8 hours. Cilia in yellow box are enlarged. Scale bar, 10 μm.

(E) 7 of 22 defective cilia lost both middle and distal ciliary segments.

(F) 15 of 22 defective cilia with distal segments shorter than 1.5 μm. m.s., middle segment; d.s., distal segment. Scale bar, 2 μm.

**Figure S4. PRP-8 remains stable in the absence of TIR1**

(A-B) Localization of PRP-8 in the absence (A) or presence (B) of 4 mM auxin for 24 hours without endogenous TIR1 expression. Phasmid cilia are indicated in gray boxes. Scale bar, 10 μm.

(C) Enlarged phasmid cilia indicated in (A).

(D) Enlarged phasmid cilia indicated in (B).

(E) Quantification of normalized PRP-8 intensity in the nuclei in IAA (-) group or IAA (+) group. Strain without TIR1 is used in this experiment. The mean intensity of IAA (-) group is normalized to 100%. *n* indicates the number of nuclei. Error bars, SD.

(F) Pe­­rcentage of abnormal cilia in IAA (-) group or IAA (+) group. *n* indicates the number of cilia. ns denotes *P* > 0.05 via Fisher’s exact test.

**Figure S5. Statistics of PRP-8 intensities**

(A) Quantification of cilia length in IAA (-) group or IAA (+) group. Normal or abnormal cilia in each group correspond to Figure 2 N. *n* indicates the number of cilia.

(B) Quantification of all nuclear PRP-8 intensities in IAA (-) group or IAA (+) group. The mean intensity of IAA (-) group is normalized to 100%. *n* indicates the number of nuclei.

(C) Quantification of all ciliary basal PRP-8 intensities in IAA (-) group or IAA (+) group. The mean intensity of IAA (-) group is normalized to 100%. For each worm, two cilia from different phasmids are counted. *n* indicates the number of cilia.

(D) Pe­­rcentage of abnormal cilia in nuclear PRP-8 (+) group or PRP-8 (-) group. Cilia with nuclear PRP-8 intensities > 0.5 belong to PRP-8 (+) group; Cilia with nuclear PRP-8 intensities < 0.3 belong to PRP-8 (-) group.

(E) Quantification of cilia length in nuclear PRP-8 (+) group or PRP-8 (-) group.

(F) Pe­­rcentage of abnormal cilia in ciliary basal PRP-8 (+) group or PRP-8 (-) group. Cilia with basal PRP-8 intensities > 0.5 belong to PRP-8 (+) group; Cilia with basal PRP-8 intensities < 0.3 belong to PRP-8 (-) group. *****P* < 0.0001 and ns denotes *P* > 0.05 via Fisher’s exact test.

(G) Quantification of cilia length in ciliary basal PRP-8 (+) group or PRP-8 (-) group. *****P* < 0.0001 and ns denotes *P* > 0.05 via unpaired *t*-test.

(H) Full image of Figure 3 B. Regenerated cilia are indicated in yellow arrows.

(I) Full image of Figure 3 G. Regenerated cilia are indicated in yellow arrows.

(J) Image of the only defective cilia after 48-hour recovery. Phasmid cilia are indicated in gray box. Scale bar, 10 μm.

(K) Enlarged phasmid cilia indicated in (C). Ciliary bases are indicated by white arrows. The ciliary tip of defective cilia is indicated by yellow arrows.

(L) Quantification of ciliary basal PRP-8 intensity before or after removal of auxin for 48 hours. Dots in black represent normal cilia, and dots in magenta represent abnormal cilia. R, recovery. *n* indicates the number of cilia. *****P* < 0.0001 and ns denotes *P* > 0.05 via unpaired *t-*test.
