# Supplementary figures and images for: Cilia regeneration requires an RNA splicing factor from the ciliary base
Source: Cell Regen. 2022 Oct 1;11:29. doi: 10.1186/s13619-022-00130-x (PMC9525525; doi:10.1186/s13619-022-00130-x)

# Figure S1

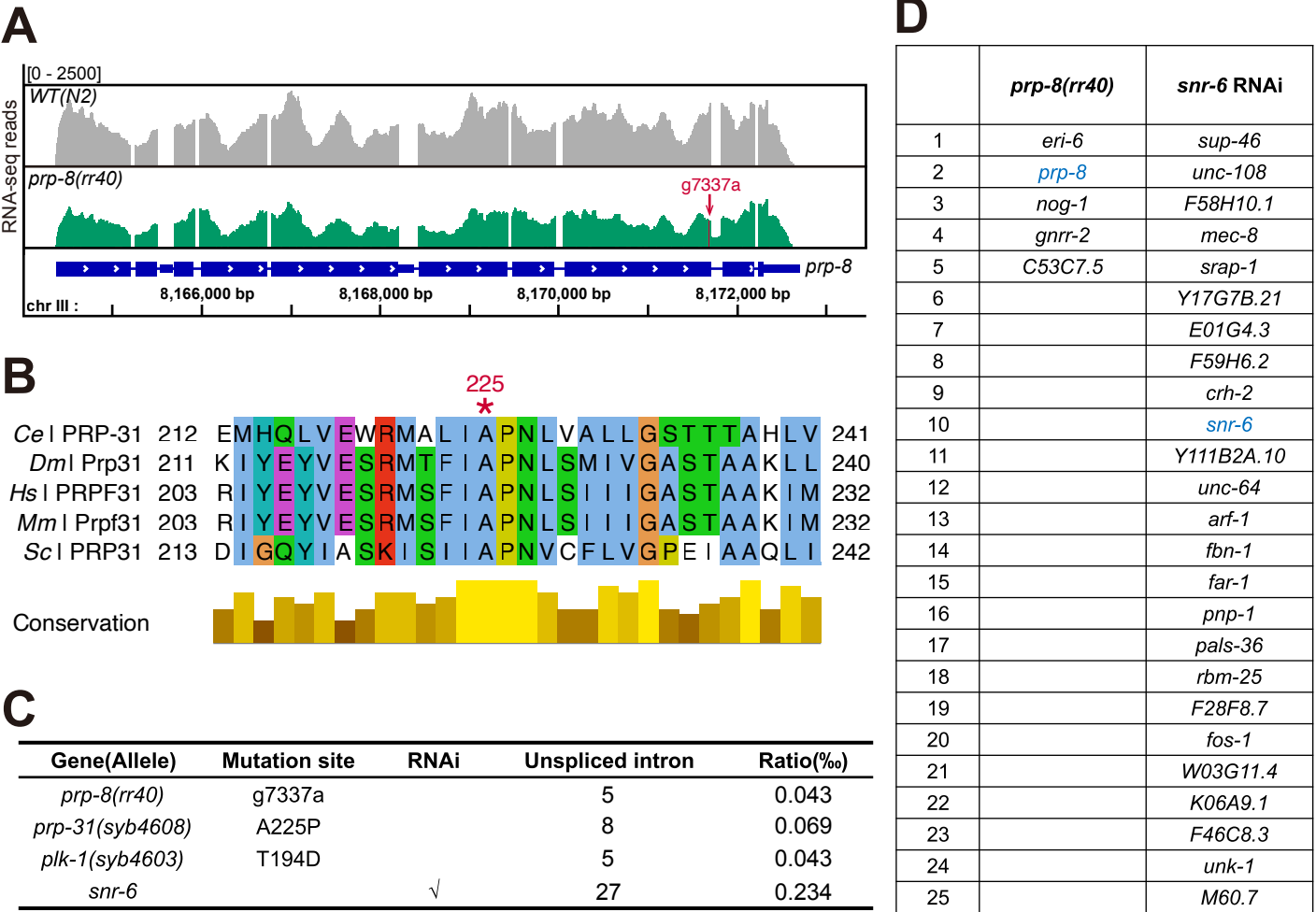

# Figure S2

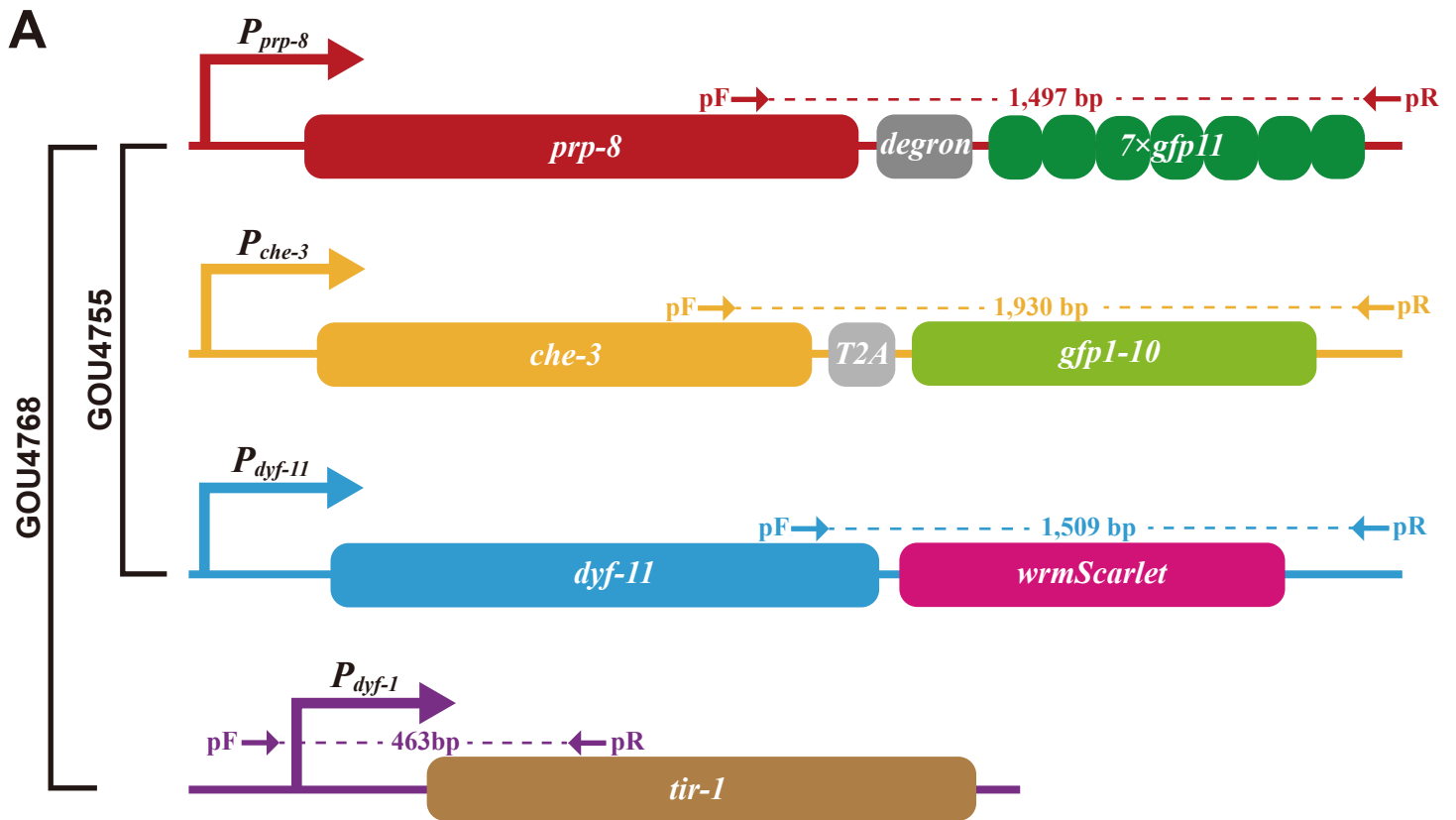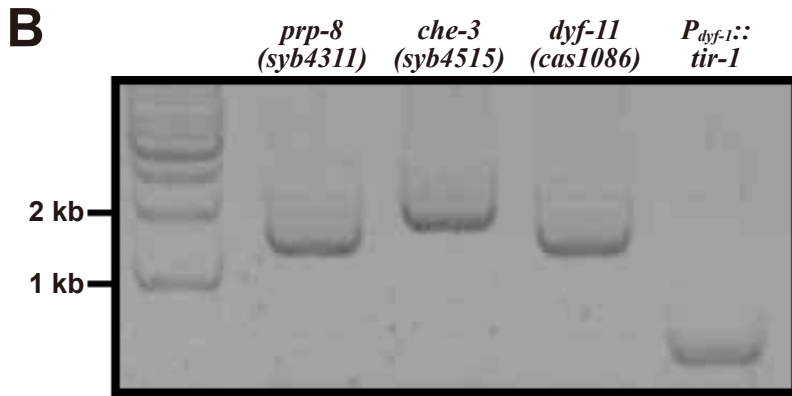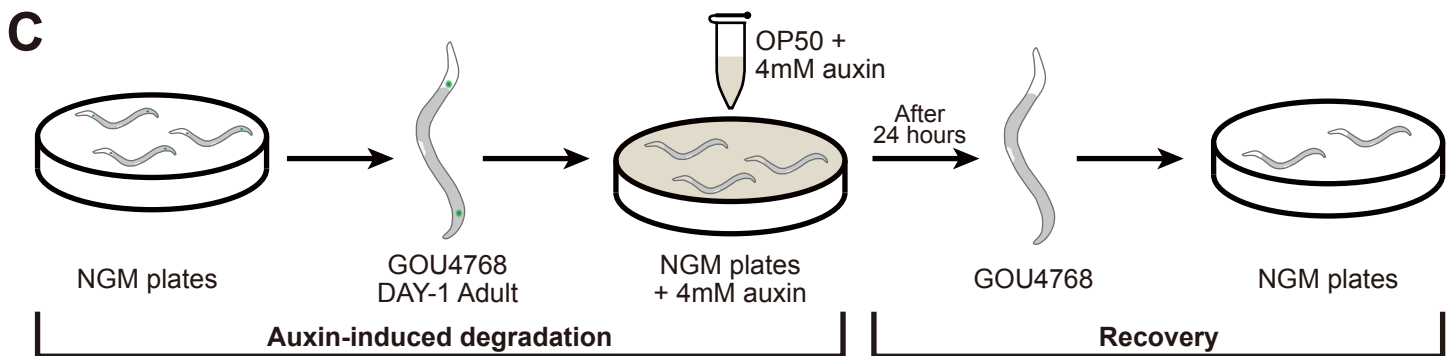

# Figure S3

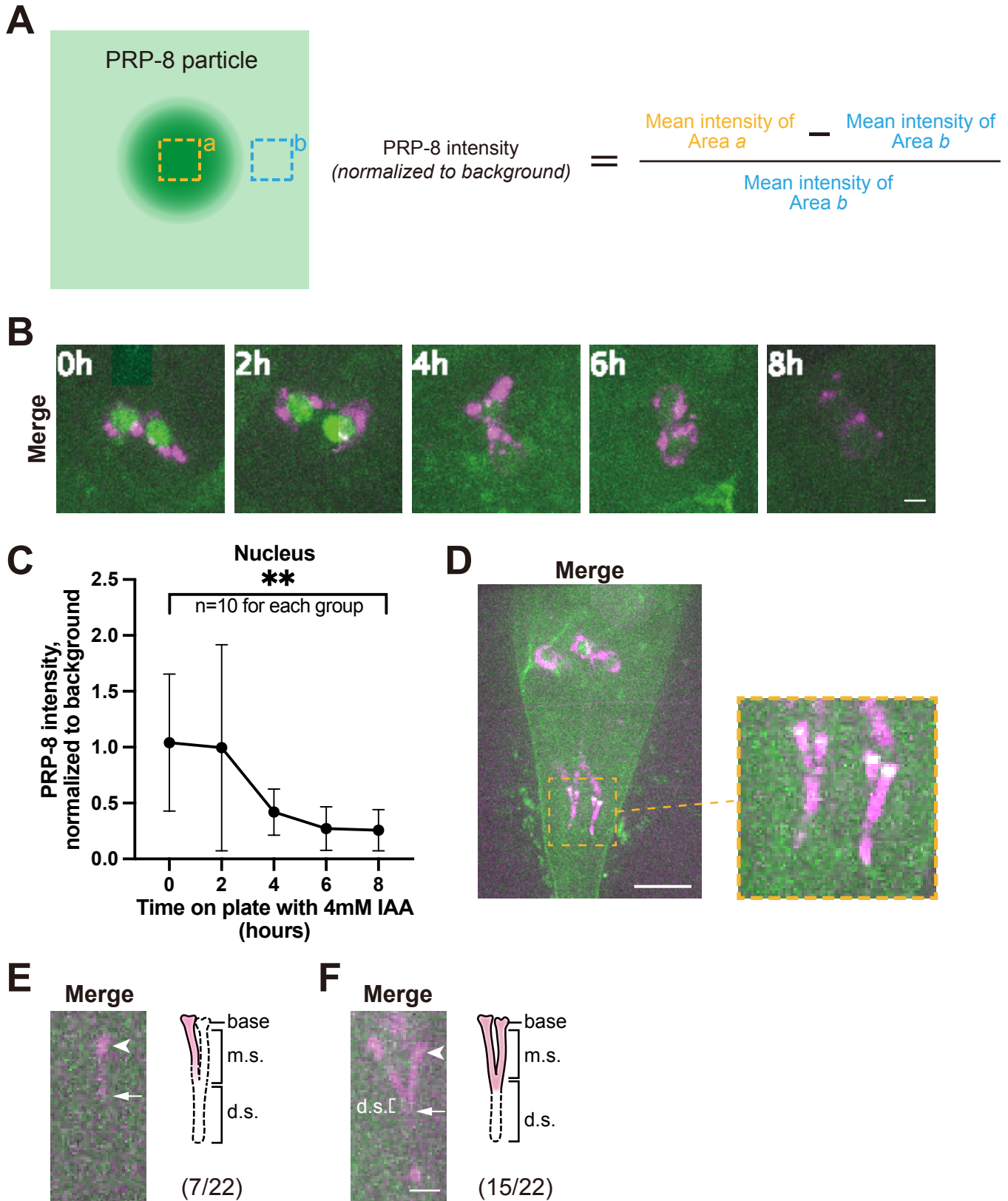

# Figure S4

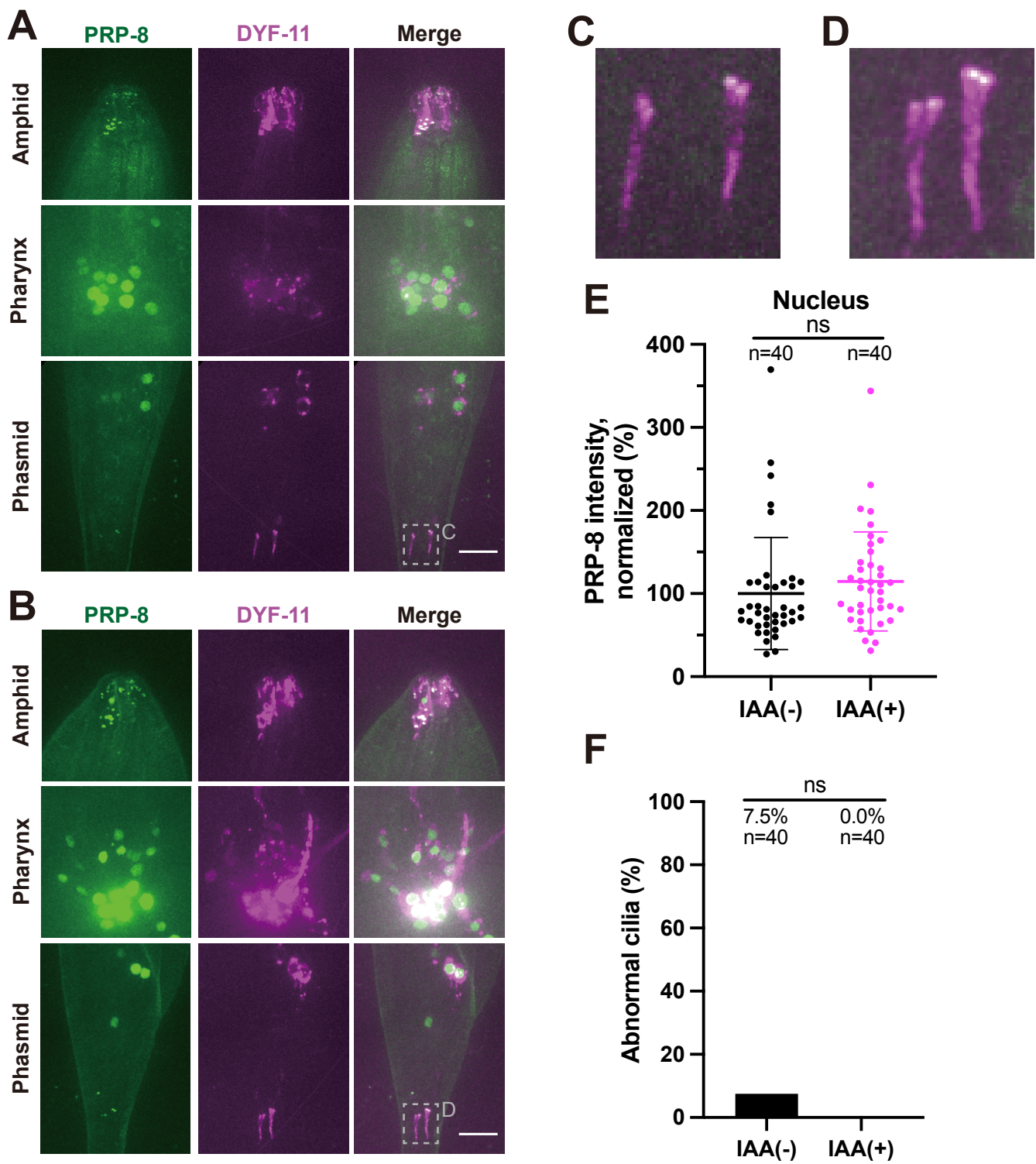

# Figure S5

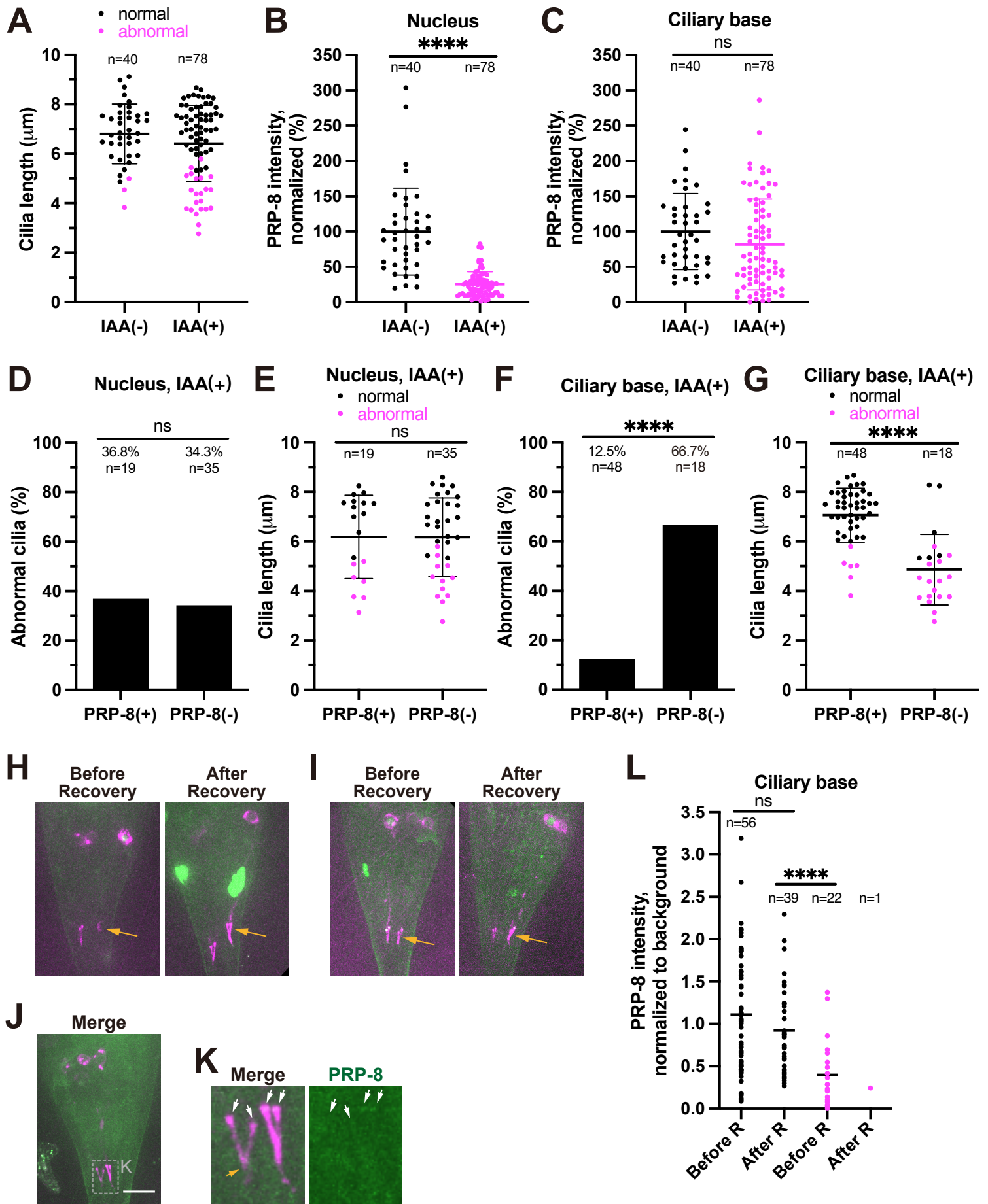

Supplement: Supplementary file 1 — Additional file 1: Fig. S1. prp-8 (rr40) weak allele has a minor effect on splicing. Fig. S2. Genetic construction of PRP-8 conditional degradation strain. Fig. S3. Gradual degradation of PRP-8 in the presence of auxin. Fig. S4. PRP-8 remains stable in the absence of TIR1. Fig. S5. Statistics of PRP-8 intensities. [file 13619_2022_130_MOESM1_ESM.zip › Xu_Figure S1-S5.pdf]
