# Supplementary material for: Cilia regeneration requires an RNA splicing factor from the ciliary base
Source: Cell Regen. 2022 Oct 1;11:29. doi: 10.1186/s13619-022-00130-x (PMC9525525; doi:10.1186/s13619-022-00130-x)
Supplement: Supplementary file 2 — Additional file 2: Table S1. C. elegans strains in this study. Table S2. Nucleotide sequences and PCR primers. [file 13619_2022_130_MOESM2_ESM.docx]

**Table S1. *C. elegans* strains in this study**

| **Strain name** | **Genotype** | **Source** |
| --- | --- | --- |
| N2 | *Wild-Type* | N.A. |
| GOU4772 | *dyf-11::wrmScarlet (cas1086) III;*  *him-5 (e1490) V.* | Microinjection |
| GOU4308 | P*dyf-1::tir-1 single copy II;*  *degron::3*gfp::che-3 I.* | Microinjection |
| PHX4311 | *prp-8::degron::7*gfp11 (syb4311) III.* | Microinjection (SunyBiotech Ltd.) |
| PHX4515 | *che-3::T2A::gfp1-10 (syb4515) I.* | Microinjection (SunyBiotech Ltd.) |
| PHX4603 | *plk-1 (syb4603) / hT2 [bli4(e937) let-? (q782) qIs48] I, III.* | Microinjection (SunyBiotech Ltd.) |
| PHX4605 | *prp-8 (rr40) (syb4605) III.* | Microinjection (SunyBiotech Ltd.) |
| PHX4608 | *prp-31 (syb4608) / hT2 [bli4(e937) let-? (q782) qIs48] I, III.* | Microinjection (SunyBiotech Ltd.) |
| GOU4596 | *osm-3 (p802) IV; zcls4 V. hsp-4::gfp.* | Microinjection |
| GOU4755 | *prp-8::degron::7*gfp11 (syb4311) III;*  *che-3::T2A::gfp1-10 (syb4515) I;*  *dyf-11::wrmScarlet (cas1086) III;*  *him-5 (e1490) V.* | Genetic cross |
| GOU4768 | *prp-8::degron::7*gfp11 (syb4311) III;*  *che-3::T2A::gfp1-10 (syb4515) I;*  *dyf-11::wrmScarlet (cas1086) III;*  P*dyf-1::tir-1 single copy II;*  *him-5 (e1490) V.* | Genetic cross |

**Table S2. Nucleotide sequences and PCR primers­**

*prp-8::degron::7*gfp11* (synonymous mutations were underlined)

forward PCR primer: AAAACCCCTTCGTTGGCTTC

reverse PCR primer: TTTCAGAGGGCACGATGT

ACACCATCGGGATATGAATGGGGTAAAGCGAATACCGACAAAGGAAATAATCCAAAGGGATATATGCCAACTCACTATGAGAAAGTGCAAATGCTTCTATCTGATCGATTCCTTGGATACTTCATGGTTCCATCAAACGGAGTGTGGAACTACAATTTCCAAGGTCAACGTTGGTCGCCTGCAATGAAGTTCGATGTGTGTCTTTCTAATCCAAAGGAGTACTATCATGAAGATCATCGGCCGGTTCACTTCCATAACTTTAAGgtattcgatctagttctaaacaattttaatatttctttcattttagGCATTCGACGATCCATTAGGAACAGGAAGCGCCGATCGCGAAGATGCATTTGCAATGCCTAAAGATCCAGCCAAACCTCCGGCCAAGGCACAAGTTGTGGGATGGCCACCGGTGAGATCATACCGGAAGAACGTGATGGTTTCCTGCCAAAAATCAAGCGGTGGCCCGGAGGCGGCGGCGTTCGTGAAGTGCCCGGGGGATCGGTGGAGCTCCACCGGTGGCGGCCGCTCTAGAGAGAATCTTTATTTTCAGGGCGCCGCCAAATTCAAAGAAACCGCTGCTGCTAAATTCGAACGCCAGCACATGGACAGCGGAGGTGGAGGTACTAGCATGCGTGACCACATGGTCCTTCATGAGTATGTAAATGCTGCTGGGATTACAGGTGGCTCTGGAGGTAGAGATCATATGGTTCTCCACGAATACGTTAACGCCGCAGGCATCACTGGCGGTAGTGGAGGACGCGACCATATGGTACTACATGAATATGTCAATGCAGCCGGAATAACCGGAGGGTCCGGAGGCCGGGATCACATGGTGCTGCATGAGTATGTGAACGCGGCGGGTATAACTGGTGGGTCGGGCGGACGAGACCATATGGTGCTTCACGAATACGTAAACGCAGCTGGCATTACTGGCGGATCAGGTGGCAGGGATCACATGGTACTCCATGAGTACGTGAACGCTGCTGGAATCACAGGCGGTAGCGGCGGTCGGGACCATATGGTCCTGCACGAATATGTCAATGCTGCCGGTATCACCTAAtcatttttcttattttatctaaaaatttattattttttctcttctaaggacctccataatttattctcattttgttgaatttcattttttggtttttttaatcactaatctatttctctcgttttttctcttcccttcgaatctcctaagcaacactgtgtgtattttcccaaaccccctcccagtccttttcaaccaaataaggttcgttctgtcagaaatttaatcctgaaattttatcattagtcttgacgtttttattcctcttttttgttctcatggtccctgaaattgtaaattgggaaatgtatgtttctcccaccttcctccactatttgttttctatataaagtgaaggtatttggtaaaacgtacgagacatctcatcatcatgaatccatttttcattccgtgttgtttcgtgt

*che-3::T2A::gfp1-10* (synonymous mutations were underlined)

forward PCR primer: AAAACCCCTTCGTTGGCTTC

reverse PCR primer: TTTCAGAGGGCACGATGT

AAAACCCCTTCGTTGGCTTCTCCAGCCGTTCAGAAAACTATTCAATCACTTGTATTTCAACAGACACCTGATGAATGGGATAGCATGTGGGCGGGACCAAGTGATCCTGCAGATTATTTAAATGTTGTTGTTAAGAAGACACGTGGAACATTGCAGgtaacagttttgaaaataaaaaataaaaaaacatgactttattgctgtcaacttaaagttggatttttcattctttggcaatatgatttgtatttcttttgtggtatttcaaattttaaaacagttttgatttttttaaaactaaatccccattccccatactccacatttaaacaaacttacaatttacacaataaatatacatttcttcagCTGTTCGAATCGTCAAAATCATCTTCTCTGCTCTCATCCCCAATTGATTTTTCGGACTTGTTCTATCCAAACATCTTTTTAAATGCACTCCGTCAAACAACATCTCGTCAAATAAAAATTCCATTAGATCAACTGATCTTATCATCAGCATGGACTCCTTCTCAACTACCAGCTAAACAATGTGTTCAAGTTCAAGGGCTCTTGCTTCAAGGAGCAACGTTTGATTCATTTCTTCGTGAAACTACAGTATCCAGTGCTGCTTATTCTCAAGCTCCTATAGTTTTTCTTGCATGGACATCTGAAAGTTCTTCAACGATTACTGGAGAGCAGATTCAGGTACCAGTGTATAGTTCAAGTGAACGATCTGATTTGATATGCTCGGTTAATATGCCATGTCGTGGTGCGGATCAATGGAATATTGCAGCTGTTGCATTGTTTTTGAGAGGTAGCGGTGAGGGCAGAGGAAGTCTGCTAACATGCGGTGACGTCGAGGAGAATCCTGGCCCAATGTCCAAAGGAGAAGAACTGTTTACCGGTGTTGTGCCAATTTTGGTTGAACTCGATGGTGATGTCAACGGACATAAGTTCTCAGTGAGAGGCGAAGGAGAAGGTGACGCCACCATTGGAAAATTGACTCTTAAATTCATCTGTACTACTGGTAAACTTCCTGTACCATGGCCGACTCTCGTAACAACGCTTACGTACGGAGTTCAGTGCTTTTCGAGATACCCAGACCATATGAAAAGACATGACTTTTTTAAGTCGGCTATGCCTGAAGGTTACGTGCAAGAAAGAACAATTTCGTTCAAAGATGATGGAAAATATAAAACTAGAGCAGTTGTTAAATTTGAAGGAGATACTTTGGTTAACCGCATTGAACTGAAAGGAACAGATTTTAAAGAAGATGGTAATATTCTTGGACACAAACTCGAATACAATTTTAATAGTCATAACGTATACATCACTGCTGATAAGCAAAAGAACGGAATTAAAGCGAATTTCACAGTACGCCATAATGTAGAAGATGGCAGTGTTCAACTTGCCGACCATTACCAACAAAACACCCCTATTGGAGACGGTCCGGTACTTCTTCCTGATAATCACTACCTCTCAACACAAACAGTCCTGAGCAAAGATCCAAATGAAAAATGAttttaattttttaaccgtgataaagttttaataaatattatttggagattgagtgattgttttattaattgattagaatatatttctaactacaacagtaatacaatacaccaaacttgcggcgctttggaaatcgagtccctgccttctccagaagtcaagagaatacaaataattatttagacgtaaccacgtagaatttgtaacattgataacgaaaatttcaaaaatccagcatcattatttttttcaatagaagacaacaatggaatgtgcaattttgtaagttttgtgctcaatctcagttctatgtgcaactatgtgaacaagttatttaagcttgctctacatatcaaatttgtggggcgttttctgggcgagaggcgggcggacatcgtgccctctgaaa

*dyf-11::wrmScarlet* (synonymous mutations were underlined)

forward PCR primer: ACAACGGAAGATGCTAGGGC

reverse PCR primer: GTGTTGAACGCTCGGGAATTT

acaacggaagatgctagggctttcaaatgaaagttgttttggatgatgtattgatacataccgtattttctcactacctttgcactactttactttgacagcttatgcatatcgtgaattttttttaatcaacattttttaattggggttttttttatatttaatttcagtgcacgtaagtactattttacacataaaatcaattttttgacattacattcgttataaaaaatatatttttcagGACGACATCGAATCAATGATTAAAGAACTGGAGCGATGGAGAAGTGAGCAGCGACGGAATGAGCAAGAGGATCAGAACAAAAAAGCGGCAGGATTCGGAGACAGCAGTAGACTCTACAACATCATCGCAAACTTACAAAAAGAAATCAATGACACGAAAGAAGAATTGAGCAAGGCTAGAGGACGAGTTCTCAACAATGAAAAACGCATTCAACTATTCATTTCCAATGfTTATGGTCAGCAAGGGAGAGGCAGTTATCAAGGAGTTCATGCGTTTCAAGGTCCACATGGAGGGATCCATGAACGGACACGAGTTCGAGATCGAGGGAGAGGGAGAGGGACGTCCATACGAGGGAACCCAAACCGCCAAGCTCAAGGTCACCAAGGGAGGACCACTCCCATTCTCCTGGGACATCCTCTCCCCACAATTCATGTACGGATCCCGTGCCTTCACCAAGCACCCAGCCGACATCCCAGACTACTACAAGCAATCCTTCCCAGAGGGATTCAAGTGGGAGCGTGTCATGAACTTCGAGGACGGAGGAGCCGTCACCGTCACCCAAGACACCTCCCTCGAGGACGGAACCCTCATCTACAAGGTCAAGCTCCGTGGAACCAACTTCCCACCAGACGGACCAGTCATGCAAAAGAAGACCATGGGATGGGAGGCCTCCACCGAGCGTCTCTACCCAGAGGACGGAGTCCTCAAGGGAGACATCAAGATGGCCCTCCGTCTCAAGGACGGAGGACGTTACCTCGCCGACTTCAAGACCACCTACAAGGCCAAGAAGCCAGTCCAAATGCCAGGAGCCTACAACGTCGACCGTAAGCTCGACATCACCTCCCACAACGAGGACTACACCGTCGTCGAGCAATACGAGCGTTCCGAGGGACGTCACTCCACCGGAGGAATGGACGAGCTCTACAAGTGAtctttcagttttccctattcagaaggagattttcattttattgaattaaatgaaaagataaacaaataaattattttatattacttagagctttgatacaatgttacaaattcgaatgaaaattgaagttgcaccaatatgttgttgtaactaaaactaaaactaaaatgtaactaaaatgttgtaactaacaaaatgttttgtttaggtccaacactatttcttattggagacaaagtggaggaaatttccaatgccactccttagctaattaattattaattgtctaaaaagtccttttcaggattagttttaaattcccgagcgttcaacac

P*dyf-1::tir1*

forward PCR primer: ACTTGCCAAATGGATTCACAGAA

reverse PCR primer: CACCAACGCTCGATCTCGTA

ttgactgtcgactcttggaagcacaatttttgatgtttcttctcatctactattaaaaataaaaataaaaaaacactgcgaattaaataaaaaactaccgtactcttttgaagacatattttaaaaacttgccaaatggattcacagaataaattaacactagaaaatgattactgaacgaaattctagcaaaactaatgctaaaactcactttttaaaaactatctgcagaaaaatatgtttggcctaaaatttcaaatatagctccattgggctctccatctgctcaaaacacacaggctcccccttcttcagtgtctcttcatctatacactattttctgtttcttcgggttaccatggatattattatgctgacactagttgaacgggagggttcagtgctcatattttgatacttatcactattttgctatatttctgtgtaagcttgtcaaaATGCAAAAGAGAATCGCCTTGTCGTTTCCAGAAGAAGTACTCGAGCACGTCTTCTCCTTCATCCAACTCGACAAGGACCGTAACTCCGTCTCCCTCGTCTGCAAGTCCTGGTACGAGATCGAGCGTTGGTGCCGTCGTAAGGTCTTCATCGGAAACTGCTACGCCGTCTCCCCAGCCACCGTCATCCGTCGTTTCCCAAAGGTCCGTTCCGTCGAGCTCAAGGGAAAGCCACACTTCGCCGACTTCAACCTCGTCCCAGACGGATGGGGAGGATACGTCTACCCATGGATCGAGGCCATGTCCTCCTCCTACACCTGGCTCGAGGAGATCCGTCTCAAGCGTATGGTCGTCACCGACGACTGCCTCGAGCTCATCGCCAAGTCCTTCAAGAACTTCAAGGTCCTCGTCCTCTCCTCCTGCGAGGGATTCTCCACCGACGGACTCGCCGCCATCGCCGCCACCTGCCGTAACCTCAAGGTAAGTTTAAACATATATATACTAACTAACCCTGATTATTTAAATTTTCAGGAGCTCGACCTCCGTGAGTCCGACGTCGACGAGGTCTCCGGACACTGGCTCTCCCACTTCCCAGACACCTACACCTCCCTCGTCTCCCTCAACATCTCCTGCCTCGCCTCCGAGGTCTCCTTCTCCGCCCTCGAGCGTCTCGTCACCCGTTGCCCAAACCTCAAGTCCCTCAAGCTCAACCGTGCCGTCCCACTCGAGAAGCTCGCCACCCTCCTCCAACGTGCCCCACAACTCGAGGAGCTCGGAACCGGAGGATACACCGCCGAGGTCCGTCCAGACGTCTACTCCGGACTCTCCGTCGCCCTCTCCGGATGCAAGGTAAGTTTAAACAGTTCGGTACTAACTAACCATACATATTTAAATTTTCAGGAGCTCCGTTGCCTCTCCGGATTCTGGGACGCCGTCCCAGCCTACCTCCCAGCCGTCTACTCCGTCTGCTCCCGTCTCACCACCCTCAACCTCTCCTACGCCACCGTCCAATCCTACGACCTCGTCAAGCTCCTCTGCCAATGCCCAAAGCTCCAACGTCTCTGGGTCCTCGACTACATCGAGGACGCCGGACTCGAGGTCCTCGCCTCCACCTGCAAGGACCTCCGTGAGCTCCGTGTCTTCCCATCCGAGCCATTCGTCATGGAGCCAAACGTCGCCCTCACCGAGCAAGGACTCGTCTCCGTCTCCATGGGATGCCCAAAGCTCGAGTCCGTCCTCTACTTCTGCCGTCAAATGACCAACGCCGCCCTCATCACCATCGCCCGTAACCGTCCAAACATGACCCGTTTCCGTCTCTGCATCATCGAGCCAAAGGCCCCAGACTACCTCACCCTCGAGCCACTCGACATCGGATTCGGAGCCATCGTCGAGCACTGCAAGGACCTCCGTCGTCTCTCCCTCTCCGGACTCCTCACCGACAAGGTCTTCGAGTACATCGGAACCTACGCCAAGAAGATGGAGATGCTCTCCGTCGCCTTCGCCGGAGACTCCGACCTCGGACTCCACCACGTCCTCTCCGGATGCGACTCCCTCCGTAAGCTCGAGATCCGTGACTGCCCATTCGGAGACAAGGCCCTCCTCGCCAACGCCTCCAAGCTCGAGACCATGCGTTCCCTCTGGATGTCCTCCTGCTCCGTCTCCTTCGGAGCCTGCAAGCTCCTCGGACAAAAGATGCCAAAGCTCAACGTCGAGGTCATCGACGAGCGTGGAGCCCCAGACTCCCGTCCAGAGTCCTGCCCAGTCGAGCGTGTCTTCATCTACCGTACCGTCGCCGGACCACGTTTCGACATGCCAGGATTCGTCTGGAACATGGACCAAGACTCCACCATGCGTTTCTCCCGTCAAATCATCACCACCAACGGACTC
